# A four-compound remedy AGILe protected H9c2 cardiomyocytes against oxygen glucose deprivation *via* targeting the TNF-α/NF-κB pathway: Implications for the therapy of myocardial infarction

**DOI:** 10.3389/fphar.2023.1050970

**Published:** 2023-01-13

**Authors:** Xiuying Zhang, Qilei Chen, Jia Zhao, Wei Zhao, Ni Fan, Yu Wang, Hubiao Chen, Jianhui Rong

**Affiliations:** ^1^ School of Chinese Medicine, Li Ka Shing Faculty of Medicine, The University of Hong Kong, Pokfulam, Hong Kong SAR, China; ^2^ School of Chinese Medicine, Hong Kong Baptist University, Pokfulam, Hong Kong SAR, China; ^3^ Department of Pharmacology and Pharmacy, Li Ka Shing Faculty of Medicine, The University of Hong Kong, Pokfulam, Hong Kong SAR, China

**Keywords:** myocardial infarction, Baoyuan Decoction, TNF-α, NF-κB, Agile

## Abstract

Myocardial infarction (MI) is a highly prevalent and lethal disease worldwide. Prevention and timely recovery are critical for the control of the recurrence and heart failure in MI survivors. The present study was designed to investigate the cardioprotective activity of the herbal medicine formula Baoyuan Decoction (BYD) and identify the active compounds and molecular targets. The ethanolic BYD extract (BYDE) was prepared by water extraction and ethanol precipitation of four herbal medicines, Astragali Radix, Ginseng Radix et Rhizoma, Cinnamomi Cortex, and Glycyrrhizae Radix et Rhizoma. Initially, BYDE was validated for the cardioprotective effectiveness in a mouse model of ischemia injury and rat cardiomyocyte H9C2 cells. As results, BYDE effectively reduced infarct size from 56% to 37% and preserved cardiac functions in mouse MI model while protected H9C2 cells against oxygen glucose deprivation. Subsequent network pharmacology analysis revealed that 122 bioactive ingredients, including flavonoids and saponins from the UPLC-MS/MS profile of BYDE, might target 37 MI-related proteins, including inflammatory and apoptotic mediators (e.g., TNF, NFKB1, CASPs, TNFRSF1A, CXCL12, BCL2A1). Pathway enrichment analysis suggested that BYDE might control the cardiac inflammation *via* targeting the tumor necrosis factor-alpha (TNF-α)/nuclear factor-κB (NF-κB) pathway while the selected targets were also implicated in IL-17 signaling pathway, lipid and atherosclerosis. Consequently, adenosine, ginsenoside Rh2, isoliquiritigenin, and licochalcone A were selected to generate the four-compound mixture AGILe and validated for the inhibitory effects on the TNF-α/NF-κB pathway. The results of the present study suggested that the mixture AGILe might be a potential cardioprotective remedy against MI.

## Introduction

Myocardial infarction (MI) is a common disease with high mobility and fatality rate, representing a common health and economic challenge world-wide (Anderson and Morrow, 2017). Upon MI, the left anterior descending branch of the heart is blocked by acute thrombus so that the myocardial cells undergo ischemia and hypoxia and heart functions are largely disturbed (White and Chew, 2008). Reperfusion is critical for maintaining the link between the cellular metabolism and mitochondrial respiratory chain for energy production and also causes oxidative stress, leading to the initiation of inflammatory response, the release of cytokines (e.g., tumor necrosis factor alpha (TNF-α)) and the activation of different signaling pathways including the nuclear factor-κB (NF-κB) pathway (Ridker et al., 2000; Yellon and Baxter, 2000; Kawano et al., 2006). Indeed, heart functions are suppressed by inflammation, cardiomyocyte hypertrophy, and myocardial fibrosis, leading to heart failure, recurrent MI and death (Thygesen et al., 2007). NF-κB inhibition appeared to be beneficial to limit infarct size and improve heart functions although the controversy remains (Kis et al., 2003; Cheng et al., 2022). Different drugs and surgical procedures have been developed to treat MI while cardiac fibrosis and heart failure remain unsolved (Cahill et al., 2017; Itier and Roncalli, 2018).

On the other hand, MI was a key target disease for traditional Chinese medicine while a panel of medicinal herbs are prescribed as a formula to achieve good efficacy (Tan et al., 2021). Among several well-documented herbal medicine formulas for the treatment of cardiovascular diseases, Baoyuan Decoction (BYD) is a four ingredient formula consisting of Astragali Radix (Huangqi, HQ), Ginseng Radix et Rhizoma (Renshen, RS), Cinnamomi Cortex (Rougui, RG), and Glycyrrhizae Radix et Rhizoma (Gancao, GC) (Shu et al., 2016). Increasing evidence validated that BYD could effectively protect myocardial cells against MI-induced damage (Yuan et al., 2018). However, the chemical diversity and mechanistic elusiveness represent two major obstacles for the wide acceptance of herbal medicines as a common therapeutic strategy world-wide. Comprehensive chemical analysis of herbal medicines does not validate the identity of active compounds whereas pharmacological studies may not reveal the exact molecular mechanisms. Nevertheless, previous studies identified several cardioprotective compounds (e.g., astragaloside IV, ginsenoside Rg1, ginsenoside Rb1) from the herbal medicine formula BYD (Parikh et al., 2019; Zheng et al., 2020; Yuan et al., 2021). It was recently revealed that BYD inhibited myocardial fibrosis (MF) and prevented heart failure (HF) in a rat model of MI through regulating the AT1/P38 MAPK/TGF-β pathway (Zhang et al., 2018). Therefore, it is important to identify the active compounds from the formula BYD and elucidate the underlying mechanisms for drug discovery.

**FIGURE 1:**
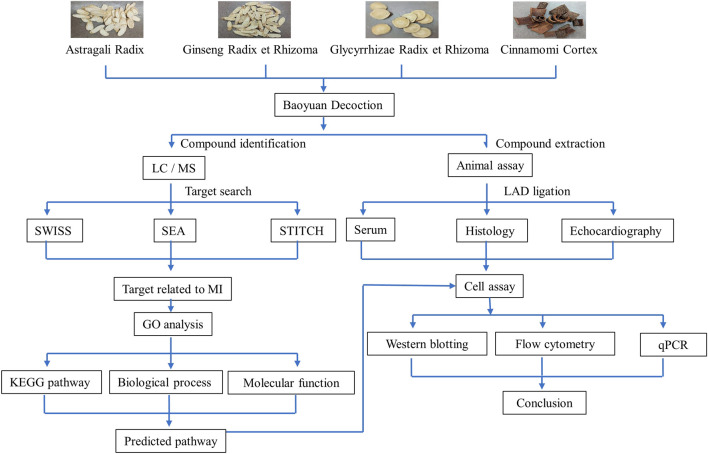
Schematic illustration of experimental strategy.

The present study was designed to perform the comprehensive chemical profiling and network pharmacology analysis of the ethanolic BYD extract (BYDE) for the active compounds and target genes with a special regard to inflammation, immune response and cardiac remodeling in MI. As outlined in Figure 1, our strategy basically involved the following eight steps: 1) To determine the cardioprotective activity of BYDE in a mouse MI model; 2) To profile the chemical composition of BYDE by UPLC-MS/MS; 3) To predict the gene/protein targets for the chemical compounds in BYDE by searching the drug-protein interaction databases (e.g., SEA, SWISS, STITCH); 4) To select MI-related protein targets at the online platform DisGeNET and CTD; 5) To reveal the drug-protein interaction network and the protein-protein interaction network; 6) To examine the effects of BYDE on the KEGG pathways, biological processes, cellular components and molecular functions; 7) To define chemical compounds for targeting the key inflammatory and apoptotic pathways; 8) To validate the activities of the mixture of the active compounds in H9c2 cells under oxygen-glucose deprivation (OGD) condition. We expected to develop a novel chemically defined and mechanism-specific remedy for the treatment of MI.

## Materials and methods

### Reagents

The dried powder of the aqueous extracts from the four herbs (i.e., HQ, RS, RG, GC) were purchased from Nong’s Pharmaceutical Ltd. (Hong Kong, China). Adenosine (CAS No. 58-61-7), ginsenoside Rh2 (CAS No. 78214-33-2), isoliquiritigenin (CAS No. 961-29-5), and licochalcone A (CAS No. 58749-22-7) were purchased from Aladdin company (Shanghai, China).

### Preparation of BYDE

BYDE was prepared by mixing four ingredients as follows: HQ, 2 g; RS, 6 g; RG, 2 g; GC, 1 g, in 55 mL water and heated at 80°C for 30 min with vortexing every 10 min. After centrifugation at 13,000 rpm for 10 min, the supernatant was collected and mixed with 495 mL of absolute ethanol to make 90% ethanol suspension. The suspension was placed in the cold-room overnight to allow precipitation to occur and centrifuged at 6,000 rpm for 30 min. The supernatant was collected, filtered, and evaporated under reduced pressure into dryness.

### UPLC-MS/MS profiling

UPLC-MS/MS profiling was performed on Agilent 6,540 Ultra High Definition (UHD) Accurate-Mass Q-TOF LC/MS system from Agilent Technologies (Santa Clara, CA, United States). The samples (2 μL) were separated on a BEH C18 UPLC column at a flow rate of .4 mL/min, column temperature at 40°C, and with a gradient from solvents of .1% formic acid in water (A) and .1% formic acid in acetonitrile B) as follows: 2%–35% B at 0–10 min, 35%–75% B at 10–18 min, and 75%–100% B at 18–22.1 min. The MS instrument was configured as follows: electrospray ionization source (ESI) for both positive and negative modes, nitrogen (N2) as drying gas, flow rate at 8 L/min, gas temperature at 300°C, nebulizer at 40 psi, sheath gas temperature at 350°C, flow rate of sheath gas at 8 L/min, capillary voltage at 3.0 kV, end plate offset at −500 V, fragmentor at 150 V, skimmer at 65 V, Oct RF Vpp at 600 V, scan range of 100–1,700 m/z, and collision energy at 25 V for MS and 45 V for MS/MS, respectively. The data were analyzed with Agilent MassHunter Qualitative Analysis B.06.00 and Agilent MassHunter Quantitative Analysis B.06.00 from Agilent Technologies (Santa Clara, CA, United States).

### Mouse model of MI

MI was induced by ligating the left anterior descending coronary artery (LAD) as previously described with modification (Molitor et al., 2021; Zhao et al., 2022). Briefly, mice were anesthetized with 100 mg/kg ketamine and 10 mg/kg xylazine by intraperitoneal injection. An incision of .5 cm was made at the left chest to expose the heart. After opening the thoracic cavity by placing the retractor, the LAD artery was visualized as a deep positioned light red vessel and ligated from 3 mm below the left origin using a 7.0 silk proline suture. The mice were exposed to ischemia for 24 h or 2 weeks. Sham control was processed by the same surgical procedure except for the ligation of LAD artery. Mice were orally treated with 2.92 g/kg BYDE every day for the treatment group, or PBS for the sham and MI group at 2 h after the surgery. Mice were subsequently examined by echocardiography imaging prior to tissue harvesting at 2 weeks after surgery.

### Assessment of infarct size

Mouse Hearts were cut into 1 cm-thick sections and stained with 2,3,5-triphenyl tetrazolium chloride (TTC) (Sigma Aldrich, Saint Louis, MO, United States) as previously described (Zhao et al., 2021). In brief, five pieces of heart sections were immersed in 2% TTC solution and incubated at 37°C for 20 min. The myocardial infarction size was analyzed with the ImageJ software (NIH, Bethesda, MD, United States).

### Histological analysis

Heart specimens were fixed with 4% paraformaldehyde for overnight and embedded with optimal cutting temperature compound (OCT) from Themo Fisher Scientific (Waltham, MA, United States). The embedded tissue samples were subsequently cut into 10 μm sections using Leica CM 1950 Cryostat microtome system and examined by terminal deoxynucleotidyl transferase dUTP nick end labeling (TUNEL) staining and H&E staining.

### Assay of serum creatine kinase (CK)/creatine kinase-MB (CK-MB)

Serum CK was measured using the creatine kinase assay kit (Cat.A032-1-1, Nanjing Jiancheng, China). CK catalyzes the conversion of creatine and ATP to phospho-creatine and ADP. The product phospho-creatine is spontaneously lysed into phosphoric acid whereas ATP and ADP are relatively stable. When ammonium molybdate was added, phosphomolybdic acid and molybdenum blue were generated for measuring the phosphomolybdic as the index of CK activity. Mouse CK-MB isoenzyme ELISA kit (Cat. H197-1-1, Nanjing Jiancheng, China) was used to determine the serum CK-MB level. Briefly, an ELISA plate was pre-coated with antibodies. Then, serum samples and recognition antigen labeled by horseradish peroxidase (HRP) was added to the microplate wells. After washing thoroughly with PBST, the retained HRP was detected by converting tetramethylbenzidine (TMB) into blue/yellow dye. The absorbance at the wavelength of 450 nm was measured for negative correlation with CK-MB concentration in the samples.

### Echocardiography

Cardiac functions were examined by echocardiography on the VisualSonics Vevo 2,100 Imaging System. Mice were anesthetized with 2 L/min isoflurane. For capturing the cardiac images, mice were subjected to 1 L/min isoflurane *via* a nose cone, imaging was performed through the parasternal short axis. The left ventricular ejection fraction (LVEF) and left ventricular fractional shortening (LVFS), left ventricular cardiac output (LVCO) and left ventricular stroke volume (LVSV) were calculated with the Vevo 2,100 analysis software.

### H9c2 cells culture and oxygen-glucose deprivation (OGD) model

Rat embryonic cardiomyocyte H9c2 cells were obtained from the American Type Culture Collection (Manassas, VA, United States) and cultured in high glucose DMEM containing 10% FBS, 100 U/mL penicillin, and 100 μg/mL streptomycin at 37°C in a humidified incubator containing 5% CO_2_. For hypoxia treatment, H9c2 cells were washed twice with PBS and incubated in glucose-free DMEM with or without the drugs. The cells were transferred to an Eppendorf Galaxy 48R hypoxia chamber (Hamburg, Germany) under the conditions of .1% O_2_ and 5% CO_2_ at 37°C for 3 h. At the end, glucose-free DMEM were removed while the cells were collected for future analysis.

### Prediction of target proteins for BYDE

The chemical compounds from BYDE were screened for the target proteins at the online platforms including STITCH 5.0 (http://stitch.embl.de/), SEA (http://sea.bkslab.org/), and SwissTargetPrediction (http://www.swisstargetprediction.ch/). The selected target proteins for BYDE were selected for MI-relatedness at the online platform DisGeNET (https://www.disgenet.org/) and CTD (http://ctdbase.org/).

### Gene ontology (GO) enrichment analysis

The GO enrichment of the target proteins was performed on the DAVID Bioinformatics Resources 6.8 platform (https://david.ncifcrf.gov/summary.jsp). The annotations of the KEGG pathway, biological process, and molecular function were downloaded for further analysis. The top 15 annotations with the highest *p*-value for each category was selected for figure generation using GraphPad Prism 8.

### Construction of the interaction network

Compound-target and target-pathway networks were generated using Cytoscape 3.9.1 (https://cytoscape.org/) to visualize the predicted interactions. In practice, protein-protein interactions were initially analyzed on the STRING platform (https://string-db.org/). The results were downloaded and visualized through Cytoscape 3.9.1. The nodes represented the compounds, proteins, and pathways, whereas edges represented the interactions.

### Western blotting analysis

Proteins were extracted from heart tissue or H9c2 cells for immunoblotting analysis. Total proteins were separated by SDS-PAGE gel and transferred to .45 μm PVDF membrane. After blocking with 5% milk solution, the membrane was incubated with primary antibodies against specific biomarker proteins at 4°C overnight. The membrane was subsequently washed and incubated with a secondary antibody. The proteins were visualized with Amersham ECL detection reagent (Cytiva, RPN2235) and analyzed with ChemiDoc XRS + imaging system from Bio-Rad (Hercules, CA, United States).

### Flow cytometric analysis

Following drug treatment, H9c2 cells were digested with EDTA-free trypsin and washed twice with PBS. Annexin V-FITC apoptosis detection kit (Vazyme, Shanghai, China) was used to detect cell apoptosis following the manufacturer’s instruction. Briefly, the cells were centrifuged at 300 × g for 5 min and resuspended in 100 μL of 1 × binding buffer. The cells were then stained with 5 μL Annexin V-FITC and 5 μL propidium iodide (PI) in the dark at room temperature for 15 min. The samples were subjected to flow cytometric analysis on NovoCyte Advanteon BVR analyzer from Agilent Technologies (Santa Clara, CA, United States).

### qRT-PCR analysis

The mRNA expression of selected biomarkers was determined by qRT-PCR technique as described (Cheng et al., 2015). Briefly, the total RNAs were isolated from the cells using TRIzol reagent (Invitrogen, CA, United States), and converted to the corresponding cDNAs using RevertAid first-strand cDNA synthesis kit from Thermo Fisher (Waltham, MA, United States). qRT-PCR was performed with specific primers from IDT (Coralville, Iowa, United States) and detection reagent SYBR Green mix (Vazyme, Shanghai, China). The primers for mouse gene were listed as follows.

TNF-α-F: 5′-TAC​TCC​CAG​GTT​CTC​TTC​AAG​G-3′
TNF-α-R: 5′-GGA​GGC​TGA​CTT​TCT​CCT​GGT​A-3′
GAPDH-F: 5′-ACT​CCC​ATT​CTT​CCA​CCT​TTG-3′
GAPDH-R: 5′-CCC​TGT​TGC​TGT​AGC​CAT​ATT-3′

### Statistical analysis

The statistical analysis was performed using One-way or Two-way ANOVA with GraphPad Prism 8 (San Diego, CA, United States). A *p*-value less than .05 was considered as statistically significant.

## Results

### *In vivo* cardioprotective effect of BYDE

**FIGURE 2:**
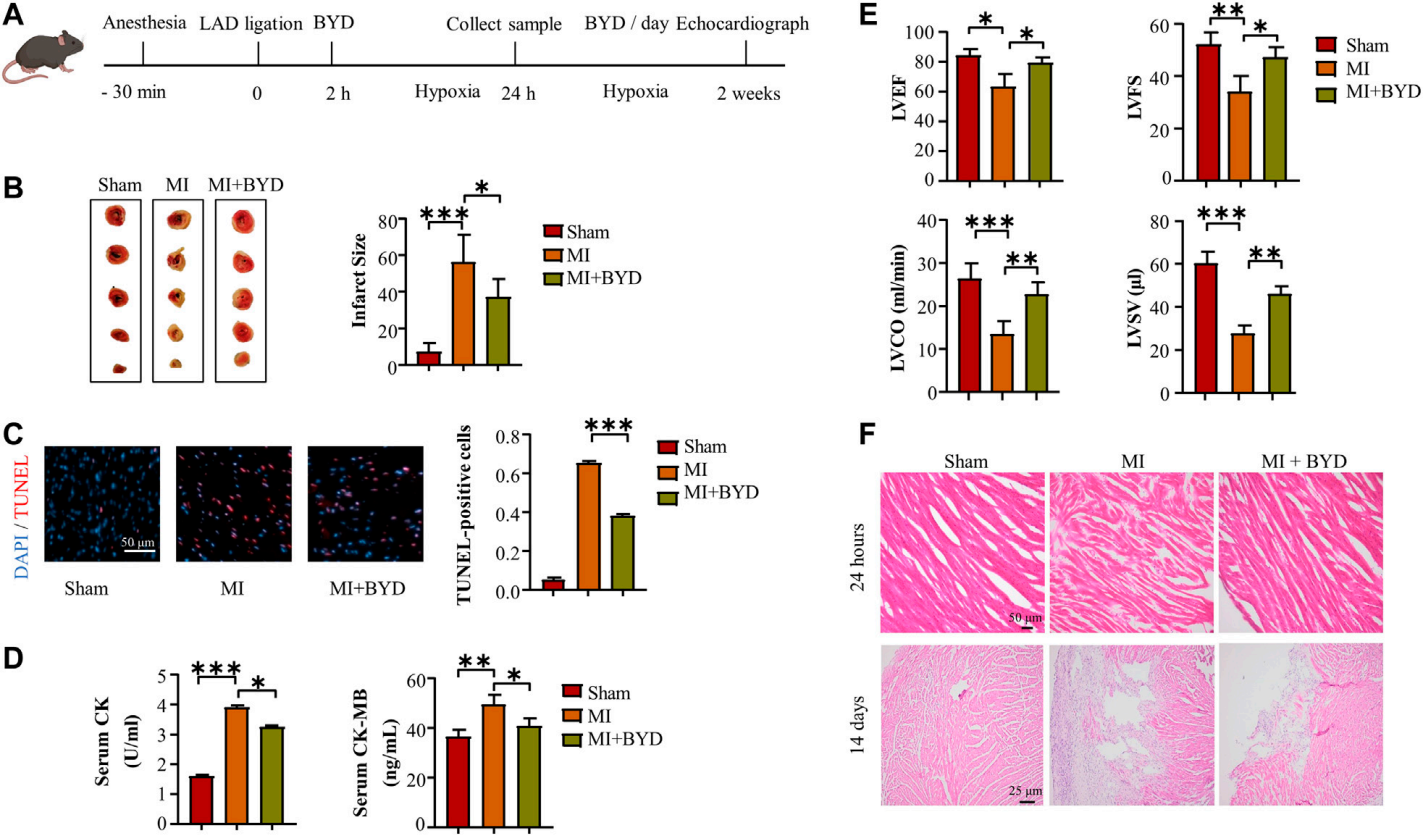
The *in vivo* cardioprotective effects of BYDE against MI. **(A)** Experimental procedure. **(B)** Infarct size in MI mice. **(C)** TUNEL Assay for DNA damage. Representative images from TUNEL staining of the myocardial layers in the risky area. Amplification, 400×; Scale bar, 50 µm. **(D)** The levels of serum CK and CK-MB. **(E)** Echocardiographic examination of heart functions. At 2 weeks after MI, M-mode echocardiography was recorded for animals in each group and calculated for left ventricular ejection fraction (LVEF) and left ventricular fractional shortening (LVFS). Doppler echocardiography. At 2 weeks after MI, PW Doppler echocardiography was recorded for animals in each group and calculated for left ventricular cardiac output (LVCO) and left ventricular stroke volume (LVSV). **(F)** Representative images from H&E staining of the myocardial layers in the risk area after 24 h ischemia challenge (top panel) and 14 days (bottom panel). Scale bar, 50 μm (top panel) and 25 μm (bottom panel). Data were expressed as mean ± S.D. (n = 6). *p < .05, **p < .01, ***p < .001.

To prepare the extract BYDE, the dried aqueous extracts of four herbs (i.e., HQ, RS, RG, GC) were mixed and subjected to extraction by warm water and precipitation with 95% ethanol. The ethanol soluble materials were evaporated to dryness, yielding the experimental BYDE preparation. The cardioprotective effect of BYDE was validated in a mouse model of MI as outlined in Figure 2A. The heart tissues were collected after treatment with BYDE (2.92 g/kg) or PBS for 24 h. Firstly, the infarct size was measured by TTC staining. As shown in Figure 2B, the mouse MI model showed severe infarction, whereas BYDE treatment markedly decreased the infarct size compared with the MI group (*p* < .05). Secondly, oxidative DNA damage in the heart tissues was detected by TUNEL assay. As shown in Figure 2C, BYDE profoundly decreased MI-induced DNA damage (*p* < .001). Thirdly, the serum CK and CK-MB were detected to estimate the extent of the myocardial injury. As shown in Figure 2D, serum CK and CK-MB were dramatically increased in the MI group whereas BYDE effectively decreased the release of CK and CK-MB into the blood (*p* < .05). Fourthly, to verify the long-term cardioprotective of BYDE, MI mice were treated with BYDE or PBS for 2 weeks by daily gavage while Sham mice received PBS. Left ventricular function was monitored by recording echocardiography. Left ventricular ejection fraction (LVEF), left ventricular fractional shortening (LVFS), Left ventricular cardiac output (LVCO) and left ventricular stroke volume (LVSV) were calculated for the amount of oxygenated blood that heart pumps to the body. The results in Figure 2E showed that BYDE treatment significantly recovered heart functions against MI-induced damage (BYDE vs. PBS, *p* < .01). The mouse heart tissue was examined by the H&E staining method. The result in Figure 2F showed that the infiltration of inflammatory cells into the influenced tissue after 24 h’ ligation was decreased when treated with BYDE. 14 days of treatment with BYDE also necessarily reduced the tissue fibrosis and scar area.

### *In vitro* cardioprotective effect of BYDE against oxygen-glucose deprivation (OGD) challenge

**FIGURE 3:**
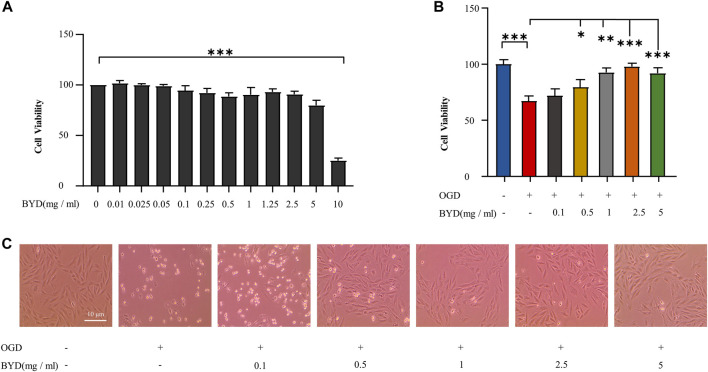
*In vitro* evaluation of BYDE for the cytoprotective effect against OGD challenge. **(A)** Effect of BYDE on the cell viability of H9c2 cells. Following BYDE treatment, the cell viability of H9c2 cells (n = 5) was examined by MTT assay. **(B)** Cytoprotective effect of BYDE on the viability of H9c2 cells against OGD challenge. Upon BYDE pretreatment and OGD challenge, the viability of H9c2 cells (n = 5) was determined by CCK-8 assay. **(C)** Effect of BYDE on the morphology of H9c2 cells against OGD challenge. Upon BYDE pretreatment and OGD challenge, the morphology of H9c2 cells (n = 3) was examined under a microscope.

To verify the *in vitro* cytoprotective effect of BYDE, H9c2 cells were pre-treated with BYDE and challenged by OGD to mimic MI. Firstly, H9c2 cells were treated with BYDE at different concentrations of up to 10 mg/mL. The cell viability was examined by MTT assay. The results in Figure 3A suggested that BYDE at the sub-toxic concentrations of up to 5 mg/mL would not alter the cell viability under normal cell culture conditions. Secondly, the cytoprotective effects of BYDE on H9c2 cells against OGD challenge were evaluated by CCK-8 assay. As shown in Figure 3B, BYDE increased the viability of H9c2 cells under OGD condition in a concentration-dependent manner. Thirdly, following BYDE treatment, the morphology of H9c2 cells was examined under a microscope. As shown in Figure 3C, OGD challenge dramatically altered the morphology of H9c2 cells whereas BYDE improved the cell morphology in a concentration-dependent manner.

### UPLC-MS/MS profiling of the chemical compounds from the preparation BYDE

**FIGURE 4:**
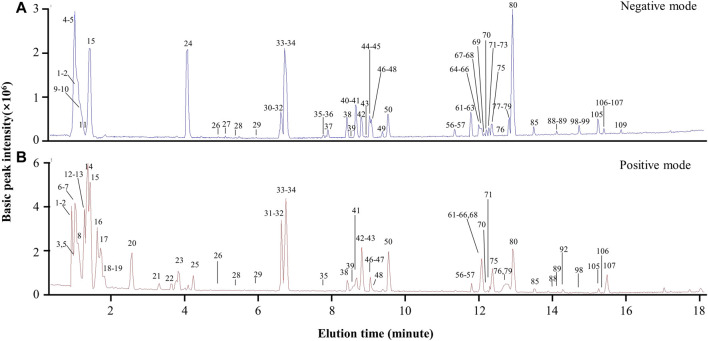
UPLC-MS/MS profiling of the preparation BYDE. **(A)** Base peak chromatogram (negative mode) of the preparation BYDE. Major peaks of BYDE were shown. **(B)** Base peak chromatogram (positive mode) of the preparation BYDE. The peaks were detected by Agilent 6,540 Ultra High Definition (UHD) Accurate-Mass Q-TOF LC/MS and numbered in order according to the retention time.

The chemical composition of the preparation BYDE was profiled by UPLC-MS/MS technology. The negative and positive ion chromatograms in Figure 4 revealed 122 compounds from the preparation BYDE while the detailed assignments were listed in Table 1. Of the compounds detected by UPLC-MS/MS technology, 43 compounds were from GC, 22 compounds from HQ, and 32 from RS while none was from RG, partly due to the limited amount of RG in the preparation BYDE or the less ionization for mass spectrometry. In fact, flavonoids and saponins were detected as the major components of BYDE. Specifically, most of the flavonoids were from GC and HQ while most of the saponins were from RS.

**TABLE 1:** UPLC MS/MS-based identification of the main compounds in BYDE.

Peak number	Compound name	Molecular formula	Molecular weight	MS/MS ions	
				Positive mode	Negative mode
1	Protonated arginine	C_6_H_14_N_4_O_2_	174.2	173.1052	175.1191
2	(2S)-2-Ammonio-4-(carbamimidamidooxy)butanoate	C_5_H_12_N_4_O_3_	176.17	175.0847	177.0985
3	Argininyl-fructosyl-glucose	C_18_H_34_N_4_O_12_	498.48		499.2249
4	D-(−)-Arabinose	C_5_H_10_O_5_	150.13	195.0523	
5	(2S)-4-Amino-2-ammonio-4-oxobutanoate	C_4_H_8_N_2_O_3_	132.12	131.0471	133.0609
7	(2S)-Pyrrolidin-1-ium-2-carboxylate	C_5_H_9_NO_2_	115.13		116.0704
10	Beta-Maltose	C_12_H_22_O_11_	342.3	387.1158	
14	Nicotinic acid	C_6_H_5_NO_2_	123.11		124.0392
16	Adenosine	C_10_H_13_N_5_O_4_	267.2400	312.0952	268.1046
24	Paeonol isomer	C_9_H_10_O_3_	166.17	165.0543	
26	Liquiritigenin-O-diglucoside^a^	C_27_H_32_O_14_	580.5300	625.1780	603.1668
26	5-Dihydroxy-liquiritigenin-O-diglucoside	C_32_H_40_O_18_	712.6500	711.2142	
26	8-Dihydroxy-liquiritigenin-O-diglucoside	C_32_H_40_O_18_	712.6500	711.2142	
28	Vicenin-2	C_27_H_30_O_15_	594.5200	593.1510	595.1662
28	Apigenin-6,8-di-C-β-d-glucopyranoside	C_27_H_30_O_15_	594.5200	593.1510	595.1662
29	Isoschaftoside	C_26_H_28_O_14_	564.4900	563.1404	565.1556
30	Liquiritigenin-O-diglucoside	C_27_H_32_O_14_	564.4900	579.1708	581.1870
31	Calycoxin-7-O-β-D-diglucoside^a^	C_22_H_22_O_10_	446.4000	491.1202	447.1292
32	5-Hydroxy-liquiritin	C_21_H_22_O_10_	434.3900	433.1144	457.1107
32	8-Hydroxy-liquiritin	C_21_H_22_O_10_	434.3900	433.1144	457.1107
33	Liquiritin apioside	C_26_H_30_O_13_	550.5100	549.1628	573.1581
34	Liquiritin	C_21_H_22_O_9_	418.3900	417.1208	441.1157
35	Pratensein^a^	C_16_H_12_O_6_	300.2600	299.0567	301.0710
35	Glicoricone	C_16_H_12_O_6_	300.2600	299.0567	301.0710
35	Kaempferol 3-O-methyl ether	C_16_H_12_O_6_	300.2600	299.0567	301.0710
35	Rhamnocitrin	C_16_H_12_O_6_	300.2600	299.0567	301.0710
36	7,4′-Dihydroxyflavone^a^	C_15_H_10_O_4_	254.2400	299.0567	
38	Isoliquiritin apioside	C_26_H_30_O_13_	550.5100	549.1623	551.1763
39	Glycyroside	C_27_H_30_O_13_	562.5200	561.1614	563.1760
39	Ononin-O-apioside	C_27_H_30_O_13_	562.5200	561.1614	563.1760
40	Licuraside	C_26_H_30_O_13_	550.5100	549.1621	
42	Ononin	C_22_H_22_O_9_	430.4000	475.1252	453.1162
43	Isoliquiritin	C_21_H_22_O_9_	418.3900	417.1197	419.1343
44	Ginsenoside Re	C_48_H_82_O_18_	947.1500	991.5484	
45	Ginsenoside Rg1	C_42_H_72_O_14_	801.0100	845.4900	823.4817
46	Licorice glycosidde B	C_35_H_36_O_15_	696.6500	695.1981	697.2130
46	Licorice glycosidde D1	C_35_H_36_O_15_	696.6500	695.1981	697.2130
46	Licorice glycosidde D2	C_35_H_36_O_15_	696.6500	695.1981	697.2130
47	Liquiritigenin	C_15_H_12_O_4_	256.2500	255.0674	257.0811
48	Licorice glycoside C2	C_36_H_38_O_16_	726.6800	725.2083	727.2229
48	Licorice glycoside A	C_36_H_38_O_16_	726.6800	725.2083	727.2229
49	9,10-Dimethoxypterocarpan-3-O-β-D-glucoside	C_23_H_26_O_10_	462.4500	507.1512	485.1420
50	Calycosin	C_16_H_1_2O_5_	284.2600	283.0625	307.0581
51	(3R)-(+)-isomucronulatol-2′-O-β-D-glucoside	C_23_H_28_O_10_	464.4600	463.1611	465.1757
52	Uralsaponin C	C_42_H_64_O_16_	824.9500	823.4118	825.4277
53	Uralsaponin F	C_44_H_64_O_19_	896.9700	895.3960	897.4121
54	Macedonoside C	C_42_H_62_O_16_	822.9300	821.3960	845.3931
55	Licorice saponin A3	C_48_H_72_O_21_	985.0700	983.4479	985.4644
56	Ginsenosdie Rf	C_42_H_72_O_14_	801.0100	845.4893	823.4809
57	Uralsaponin M	C_44_H_64_O_18_	880.9700	879.4013	881.4169
57	22-β-acetylglycyrrhizin	C_44_H_64_O_18_	880.9700	879.4013	881.4169
58	Uralsaponin U	C_42_H_62_O_17_	838.9300	837.3908	839.4044
58	Uralsaponin N	C_42_H_62_O_17_	838.9300	837.3908	839.4044
59	Ginsenoside Ra2	C_58_H_98_O_26_	1,211.3800	1,209.6256	1,233.6237
60	Ginsenoside Ra3	C_59_H_100_O_27_	1,241.4100	1,239.6366	1,263.6343
61	Ginsenoside Rb1	C_54_H_92_O_23_	1,109.2900	1,153.6001	1,131.5929
62	Lupenone	C_30_H_48_O	424.7000		425.3779
63	Isoliquiritigenin	C_15_H_12_O_4_	256.2500	255.0670	257.0811
64	Ginsenoside (S)-Rg2	C_42_H_72_O_13_	785.0100	829.4956	807.4863
65	Ginsenoside Rc	C_53_H_90_O_22_	1,079.2600	1,123.5903	1,101.5815
66	Ginsenoside Ra1	C_58_H_98_O_26_	1,211.3800	1,209.6276	1,233.6229
67	Ginsenoside Rh1	C_36_H_62_O_9_	638.8700	683.4373	
68	Formononetin	C_16_H_12_O_4_	268.2600	267.0674	291.0630
69	Ginsenoside (R)-Rg2	C_42_H_72_O_13_	785.0100	829.4945	
70	Ginsenoside Ro	C_48_H_76_O_19_	957.1000	955.4910	979.4870
71	Ginsenoside Rb2	C_53_H_90_O_22_	1,079.2600	1,123.5903	1,101.5820
72	Ginsenoside F1	C_36_H_62_O_9_	638.8700	683.4371	
73	Yunganoside E2	C_42_H_60_O_16_	820.9100	819.3797	
74	Astrapterocarpan	C_17_H_16_O_5_	300.3100		301.1070
75	Licorice saponin G2	C_42_H_62_O_17_	838.9300	837.3921	861.3886
76	Astragaloside III	C_41_H_68_O_14_	784.9700	829.4581	807.4504
77	Ginsenoside RS2	C_55_H_92_O_23_	1,121.3000	1,165.5989	
78	Astragaloside IV	C_41_H_68_O_14_	784.9700	829.4581	
79	Ginsenoside Rd	C_48_H_82_O_18_	947.1500	991.5486	969.5395
80	Glycyrrhizic acid	C_42_H_62_O_16_	822.9300	821.3973	823.4123
81	Ginsenoside RS1	C_55_H_92_O_23_	1,121.3000	1,165.5989	
82	Glycycoumarin	C_21_H_20_O_6_	368.3800	367.1191	
82	Isoglycycoumarin	C_21_H_20_O_6_	368.3800	367.1191	
82	Licoarylcoumarin	C_21_H_20_O_6_	368.3800	367.1191	
82	Topazolin	C_21_H_20_O_6_	368.3800	367.1191	
82	7-O-Methylluteone	C_21_H_20_O_6_	368.3800	367.1191	
83	Astragaloside II	C_43_H_70_O_15_	827.0000	871.4681	
84	22-Dehydroxyl-uralsaponin C	C_42_H_64_O_15_	808.9500	807.4165	809.4318
85	Ginsenoside Ro^a^	C_48_H_76_O_19_	957.1000	955.4890	957.5051
86	Cyclogaleginoside D	C_43_H_70_O_15_	827.0000	871.4680	849.4606
87	Licorice saponin J2	C_42_H_64_O_16_	824.9500	823.4112	825.4275
88	Licoisoflavone B	C_20_H_16_O_6_	352.3400	351.0878	353.1021
88	Allolicoisoflavone B	C_20_H_16_O_6_	352.3400	351.0878	353.1021
88	Semilicoisoflavone B	C_20_H_16_O_6_	352.3400	351.0878	353.1021
90	Isostragaloside II	C_43_H_70_O_15_	827.0000	871.4678	849.4608
91	Ginsenoside Rg6	C_42_H_70_O_12_	767.0000	811.4839	
92	Glyasperin C	C_21_H_24_O_5_	356.4100	355.1554	357.1695
93	Glycyrflavoside C	C_42_H_64_O_15_	808.9500	807.4158	831.4144
93	Yunganoside I2	C_42_H_64_O_15_	808.9500	807.4158	831.4144
94	Ginsenoside F4	C_42_H_70_O_12_	767.0000	811.4828	
95	Gancaonin L	C_20_H_18_O_6_	354.3500	353.1037	355.1178
95	(Iso)licoflavonol	C_20_H_18_O_6_	354.3500	353.1037	355.1178
95	Licoisoflavanone	C_20_H_18_O_6_	354.3500	353.1037	355.1178
95	Licoisoflavone A	C_20_H_18_O_6_	354.3500	353.1037	355.1178
95	Luteone	C_20_H_18_O_6_	354.3500	353.1037	355.1178
96	Ginsenoside Rk3	C_36_H_60_O_8_	620.8500	665.4264	
97	Astragaloside I	C_45_H_72_O_16_	869.0400	913.4789	891.4716
99	Ginsenoside Rh4	C_36_H_60_O_8_	620.8500	665.4259	
100	Chikusetsusaponin IVa	C_42_H_66_O_14_	794.9600	793.4367	817.4344
101	Licochalcone A	C_21_H_22_O_4_	338.4000	337.1445	339.1594
102	Glycyrin	C_22_H_22_O_6_	382.4100	381.1353	405.1309
102	Licoricone	C_22_H_22_O_6_	382.4100	381.1353	405.1309
103	Isoastragaloside I	C_45_H_72_O_16_	869.0400	913.4782	891.4718
104	Glycyrol	C_21_H_18_O_6_	366.3600	365.1032	
104	Isoglycyrol	C_21_H_18_O_6_	366.3600	365.1032	
104	Glycyrrhiza isoflavone C	C_21_H_18_O_6_	366.3600	365.1032	
104	Hirtellanine I	C_21_H_18_O_6_	366.3600	365.1032	
105	Ginsenoside (R)-Rg3	C_42_H_72_O_13_	785.0100	829.4955	807.4869
106	Galbrocoumarin	C_20_H_16_O_5_	336.3400	335.0933	337.1076
106	Glabrone	C_20_H_16_O_5_	336.3400	335.0933	337.1076
106	Isoglabrone	C_20_H_16_O_5_	336.3400	335.0933	337.1076
106	Isoderrone	C_20_H_16_O_5_	336.3400	335.0933	337.1076
107	Ginsenoside (S)-Rg3	C_42_H_72_O_13_	785.0100	829.4957	
108	Cyclosieversioside B	C_45_H_72_O_16_	869.0400	913.4785	891.4712
110	Ginsenoside Rk1	C_42_H_70_O_12_	767.0000	811.4846	789.4762
111	Ginsenoside Rg5	C_42_H_70_O_12_	767.0000	811.4844	789.4766
112	Ginsenoside Rh2	C_36_H_62_O_8_	622.8700	667.4427	

^a^ isomer.

### Prediction of 37 target proteins for the BYDE

The chemical compounds from the BYDE were used to predict the target proteins through two major steps: 1) search for the drug-protein interactions in the online databases like SEA, SWISS, and STITCH; 2) search for MI-related target proteins at the online platform DisGeNET and CTD. From the step-1 selection, the potential targets from three databases were merged. From the step-2 selection, as shown in Table 2, 64 chemical compounds from BYDE were used to select 37 proteins that are associated with myocardial infarction with high gene-disease association scores. The list of target proteins included several MI-associated biomarkers (e.g., CASPs, CYPs, ALOXs) and multiple well-known inflammatory mediators (e.g., TNF-α, NF-κB, COX-2).

**TABLE 2:** Molecular targets for the active compounds from BYDE.

Molecular targets	Active compounds from BYDE
NOS2	Protonated arginine
ABCB1	Isoschaftoside, Calycosin, Formononetin, Licoisoflavone B, Licochalcone A, Vicenin-2, Rhamnocitrin, Licoisoflavone A, Isoderrone, Topazolin
ADORA1	Adenosine, L-Adenosine, Rhamnocitrin
ADORA3	Adenosine, L-Adenosine
BCL2A1	ginsenoside Rh2
CASP3	Formononetin
CASP9	formononetin
CREB1	Isoschaftoside, Glycycoumarin, Glycyrol, Vicenin-2, Isoglycyrol, Rhamnocitrin, Topazolin
CXCL12	Paeonol, Isoliquiritin, Licorice glycoside C2, Isoliquiritigenin, Formononetin, Licochalcone A, Licorice glycoside A
CYP3A4	Nicotinic acid, Isoliquiritigenin
ESR1	Paeonol, Liquiritin, Isoliquiritin, Liquiritigenin, Calycosin, Isoliquiritigenin, Formononetin, Glycycoumarin, Glycyrin, Glycyrol, Glicoricone, Isoglycycoumarin, Licoricone, Isoglycyrol, Rhamnocitrin, Topazolin, Liquiritigenin
FOS	Paeonol, Isoliquiritigenin
GSK3B	Formononetin
HIF1A	Glycycoumarin, Glyasperin C, Glycyrin, Glycyrol, Licoricone, Licoisoflavone A, Topazolin, Luteone, 7-O-Methylluteone
HMGB1	calycosin
HSD11B2	Uralsaponin C, Uralsaponin F, Macedonoside C, Licorice saponin A3, Uralsaponin M, Uralsaponin U, Lupenone, Ginsenoside Ro, Licorice saponin G2, Glycyrrhizic acid, Chikusetsusaponin IVa, Uralsaponin N, Isomer of Ginsenoside Ro, 22-β-acetylglycyrrhizin, Licorice saponin J2, Glycyrrhizic, Licorice saponin G2
ICAM1	paeonol
IL6	Isoschaftoside, Liquiritin, Glycyroside, Ononin, Vicenin-2
JUN	Paeonol, Isoliquiritigenin
LGALS3	Beta-Maltose, Liquiritin, Glycyroside, Ononin, Macedonoside C, Ginsenoside Rk1, Ginsenoside Rg5
MAPK3	calycosin
MMP1	Isoliquiritigenin
MMP2	Isoliquiritigenin
MMP9	Isoliquiritigenin
MPO	Rhamnocitrin
NFE2L2	Paeonol, Isoliquiritigenin, Ginsenoside Ro, Chikusetsusaponin IVa, Licochalcone A, Licorice glycoside A, Isomer of Ginsenoside Ro
NFKB1	Paeonol, Licorice glycoside C2, Isoliquiritigenin, Glycycoumarin, Licochalcone A, Glycyrin, Glycyrol, Licorice glycoside A, Licoricone, Topazolin, Luteone, 7-O-Methylluteone
NOS2	Protonated arginine (HQ1)
P2RY12	Adenosine
PLAT	Paeonol
PLAU	Paeonol
PLCG1	L-Adenosine
PTGS2	paeonol
RELA	Paeonol, Lupenone, Ginsenoside Ro, Gancaonin L, Chikusetsusaponin IVa, Licochalcone A, Glycyrin, Glycyrol, Glicoricone, Licoricone, Licoisoflavone A, Topazolin, Luteone, 7-O-Methylluteone, Isomer of Ginsenoside Ro
TNF	Ononin
TNNI3	Isoliquiritin
VCAM1	paeonol
VEGFA	Beta-Maltose, Isoschaftoside, Liquiritin apioside, Liquiritin, Isoliquiritin apioside, Glycyroside, Licuraside, Isoliquiritin, Ononin, Liquiritigenin, Macedonoside C, Licorice saponin A3, Ginsenoside Ra2, Ginsenoside Ra3, Ginsenoside Rb1, Ginsenoside Rc, Ginsenoside Ro, Ginsenoside Rd, Ginsenoside F4, Chikusetsusaponin IVa, Ginsenoside Rk1, Ginsenoside Rg5, Vicenin-2, Rhamnocitrin, Ginsenosdie Rf, Isomer of Ginsenoside Ro

**FIGURE 5:**
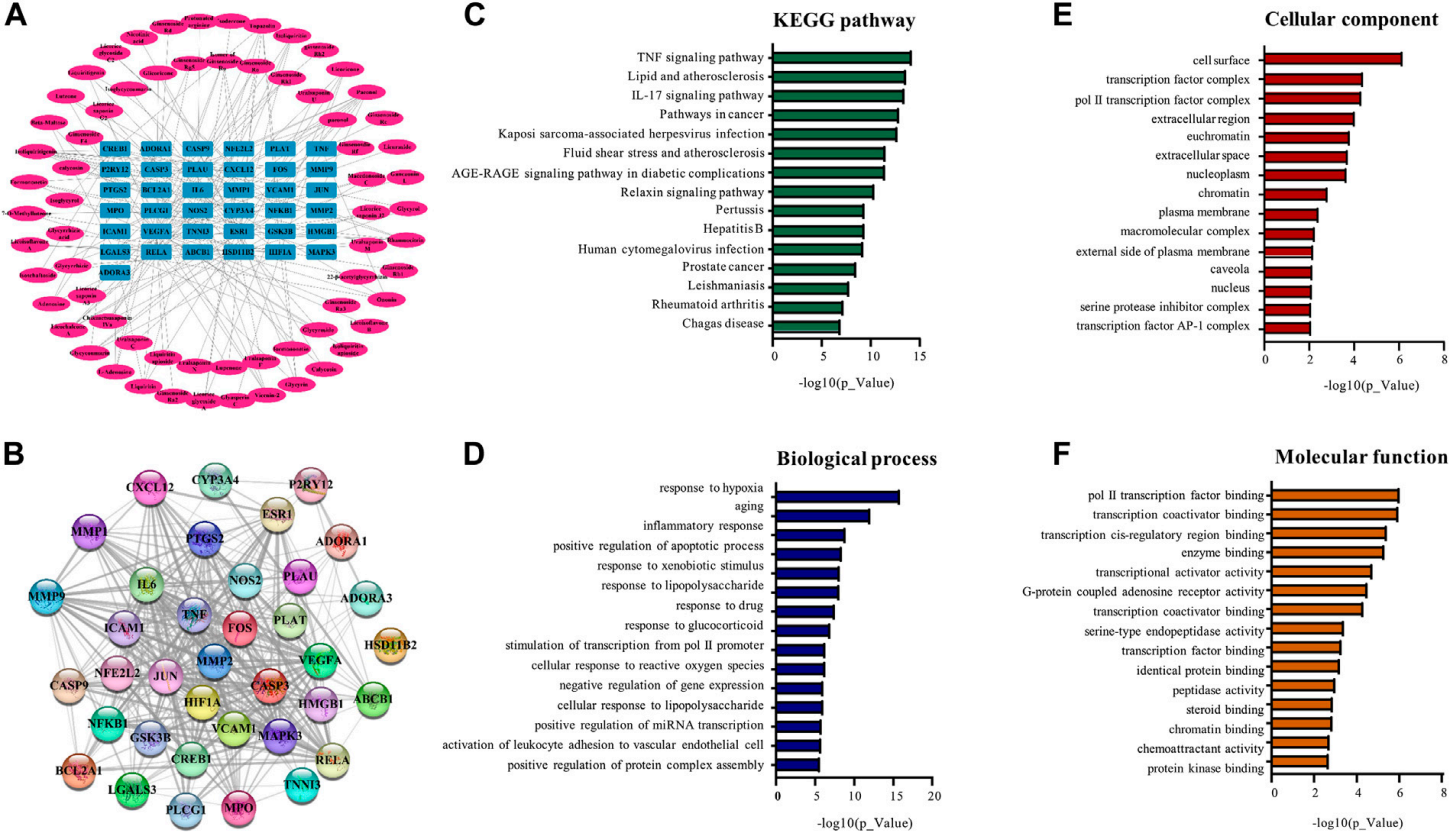
Network pharmacology analysis for the compound-target relationship and the signaling pathways. **(A)** Compound-target relationship. The outside oval shapes represent the active compounds whereas the inner rectangles represent the targets. **(B)** Protein-protein interaction network of the targets. The colored nodes represent BYDE targets whereas the edges represent the interactions between the proteins. **(C)** The top 15 signaling pathways from the KEGG analysis. **(D)** The top 15 biological process from enrichment analysis. **(E)** The top 15 cellular components from enrichment analysis. **(F)** The top 15 molecular functions from enrichment analysis.

To visualize the complex compound-target relationships, the targets were clustered at the center whereas the chemical compounds with special IDs were arranged to surround the target cluster using the software Cytoscape 3.9.1. As shown in Figure 5A, a total of 64 chemical compounds were connected with the corresponding gene targets by lines to form the compound-target network with a total of 101 nodes (including 37 targets and 64 compounds in BYDE) and 170 edges. The protein-protein interaction network was predicted for the 37 MI-related protein targets by STRING. Figure 5B consolidated the interactions between different protein targets for the regulation of inflammation (e.g., CXCL12, IL6, TNF, NFkB1, NOS2, PTGS2), apoptosis (e.g., CASP3, CASP9), cell survival (e.g., BCL2A1), angiogenesis (e.g., CREB1, ESR1, VEGFA) and cardiac remodeling (e.g., MMP1, MMP2, MMP9).

To perform pathway enrichment analysis, 37 MI-related target proteins were loaded onto the DAVID 6.8 platform. As results, the target proteins were found to be significantly associated with 112 KEGG pathways, 210 biological processes, 28 cellular components and 43 molecular functions. The top 15 significantly enriched terms in KEGG, BP, MF, and CC categories (*p* < .05, *p*-values were corrected using the Benjamini–Hochberg procedure) were listed in Figures 5C–F. BYDE appeared to be able to interact with many cellular components in the cell surface, transcription factor complex or extracellular space and regulate inflammatory response (e.g., COX-2, TNF-α and NF-κB), chemoattractant activity (e.g., CXCL), cellular response to hypoxia (e.g., HIF1A, TNF, VEGFA) and transcription factor binding (e.g., JUN, CREB1).

### Quantification of four key active compounds in BYDE

**FIGURE 6:**
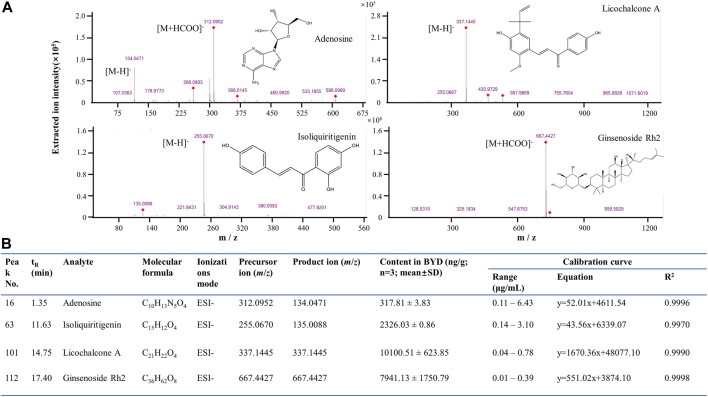
UPLC-QTOF-MS/MS quantification of the active compounds in BYDE. **(A)** UPLC-QTOF-MS/MS analysis of four standard compounds: The compounds were detected by ESI at negative ion mode. **(B)** MRM quantification results of four compounds in BYDE. Four compounds were detected by ESI at negative ion mode and quantified by MRM method whereas the corresponding standard compounds were used to generate standard curve.

To verify the network analysis results, we focused on the active compounds for targeting the TNF-α/NF-κB pathway. Five compounds (i.e., adenosine, ginsenoside Rh2, iso-liquiritigenin, licochalcone A and paeonol) were found to be associated with the TNF-α/NF-κB pathway. We subsequently employed UPLC-MS/MS technology to quantify the presence of these compounds in BYDE by the multiple reaction monitoring (MRM) method. As shown in Figure 6A Adenosine was detected with fragments in *m/z* values of 266.0893 [M-H]^-^ and 312.0952 [M + HCOO]^-^, ginsenoside Rh2 of 667.4427 [M + HCOO]^-^, iso-liquiritigenin of 255.0670 [M-H]^-^, licochalcone A of 337.1445 [M-H]^-^, whereas paeonol was not detectable in BYDE. By normalizing against the concentrations of the corresponding standard compounds (Figure 6B), the concentrations of four compounds in BYDE were calculated as follows: adenosine, 317.81 ± 3.83 ng/g; ginsenoside Rh2, 7,941.13 ± 1750.79 ng/g; iso-liquiritigenin, 2,326.03 ± .86 ng/g; licochalcone A, 10,100.51 ± 623.85 ng/g (n = 3; mean ± SD). Based on these results, four active compounds (i.e., adenosine, ginsenoside Rh2, iso-liquiritigenin, licochalcone A) were combined at the same ratio as that in BYDE to form the mixture AGILe while the concentration was normalized to the biologically effective concentration of ginsenoside Rh2 (Qi et al., 2022).

### Validation of the mixture AGILe for targeting the TNF-α/NF-κB pathway

**FIGURE 7:**
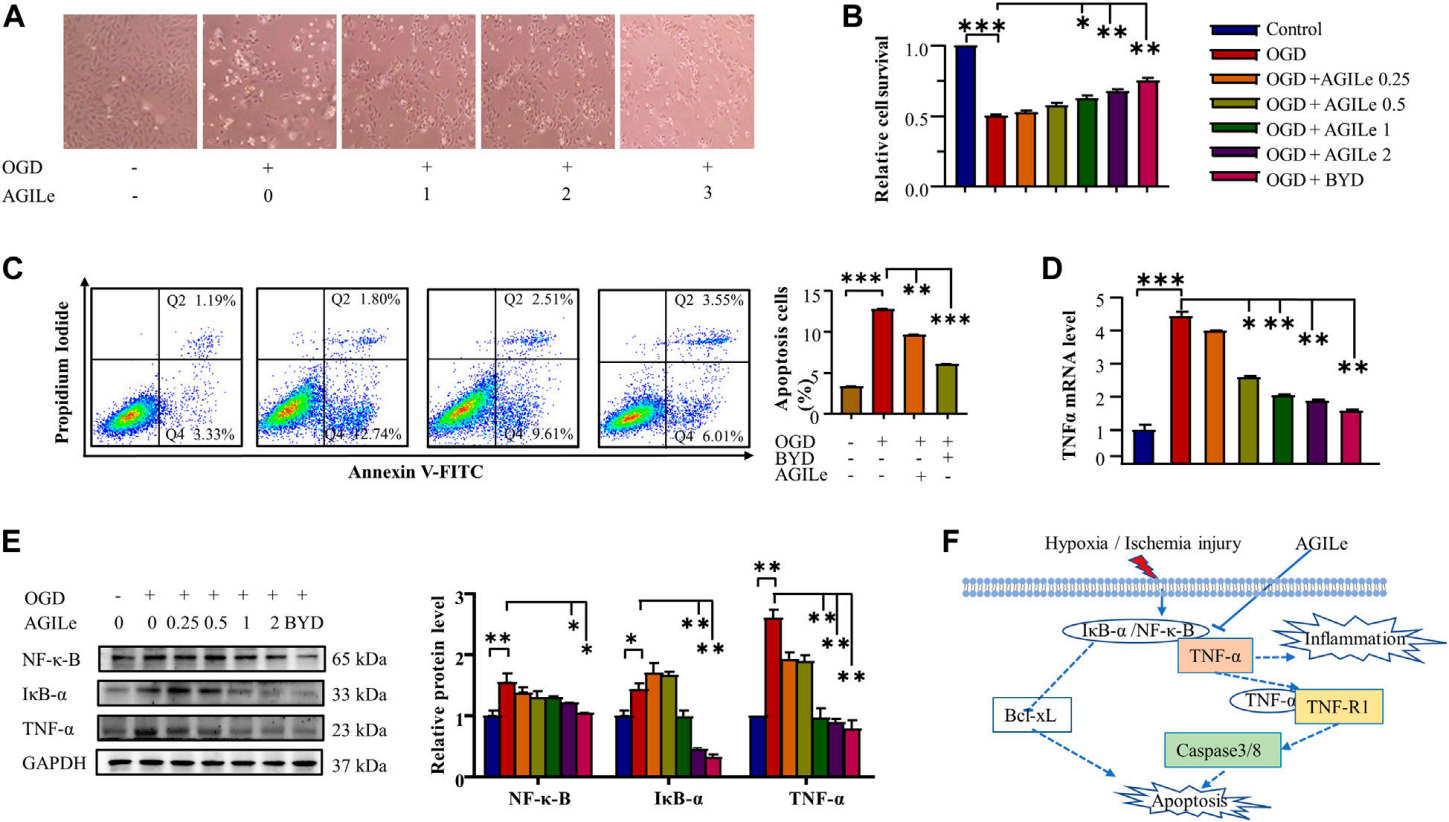
*In vitro* cardioprotective effect of the mixture AGILe. **(A)** Effect of AGILe on the morphology of H9c2 cells under OGD condition. After AGILe treatment and OGD challenge, H9c2 cells (n = 3) were examined under a microscope. **(B)** MTT assay for the viability of H9c2 cells. After AGILe/BYDE treatment and OGD challenge, H9c2 cells (n = 3) were incubated with MTT and measured on a microplate reader. **(C)** Flow cytometric analysis of apoptotic H9c2 cell. After AGILe/BYDE treatment and OGD challenge, H9c2 cells (n = 3) were stained with Annexin V-FITC and detected on a flow cytometer. **(D)** qRT-PCR quantification of TNF-α mRNA. After AGILe/BYDE treatment and OGD challenge, TNF-α mRNA (n = 3) was quantified by qRT-PCR technique. **(E)** Western blot analysis of NF-κB, IkB-α, and TNF-α. After AGILe/BYDE treatment and OGD challenge, H9c2 cells (n = 3) were lysed and analyzed by Western blotting with specific antibodies. **(F)** Molecular mechanism underlying the cytoprotection of the mixture AGILe.

The mixture AGILe was tested for the cardioprotective effect on the survival of H9c2 cells under OGD condition. In practice, H9c2 cells were pre-treated with the mixture AGILe at different concentrations, and subsequently challenged by OGD for 3 h. Firstly, the cell morphology was examined under a microscope and the representative images were shown in Figure 7A. The mixture AGILe preserved the cell morphology against OGD challenge in concentration-dependent manner. Secondly, upon AGILe treatment and OGD challenge, the viability of the H9c2 cells was determined by standard MTT assay. As shown in Figure 7B, AGILe pretreatment protected H9c2 cells against OGD challenge in concentration-dependent manner. Importantly, the mixture AGILe at 2x concentration increased the cell survival as effectively as BYDE at the concentration of 2 mg/mL. Thirdly, upon the treatment with AGILe or BYDE and the challenge of OGD, the apoptotic H9c2 cells were stained with Annexin V-FITC and assessed by flow cytometry. As shown in Figure 7C, OGD caused 12.7% of H9c2 cells to undergo apoptosis, whereas AGILe and BYDE pretreatment effectively reduced the cell apoptosis (*p* < .001). Fourthly, the mRNA level of TNF-α was determined by qRT-PCR technique. As shown in Figure 7D, OGD increased the mRNA expression level of TNF-α, whereas AGILe and BYDE pretreatment effectively reduced TNF-α mRNA level (*p* < .01). Fifthly, the protein levels of TNF-α, IkB-α and NF-κB were detected by Western blot analysis. As shown in Figure 7E, OGD induced the expression of TNF-α, IkB-α and NF-κB to certain extent, whereas AGILe and BYDE pretreatment reduced the levels of TNF-α, IkB-α and NF-κB in concentration-dependent manner (*p* < .01). These results validated the potential of the mixture AGILe in the protection of cardiomyocytes against OGD challenge and the suppression of the TNF-α/NF-κB pathway (Figure 7F).

## Discussion

The chemical complexity and polypharmacological synergy highlight that herbal medicines can be valuable resource of novel therapies against cardiovascular diseases including MI. The current study focused on identification of the bioactive compounds from the traditional formula BYD and prediction of target proteins towards development of chemically defined and mechanism-specific polychemical remedies. Traditionally, the extract BYDE was prepared by water extraction so that it might contain various water-soluble substances including inorganic minerals, oligo- and polysaccharides. The present study made a BYD preparation composed of the ethanol-soluble secondary metabolites while the cardioprotective activity of the preparation BYD was proven in a mouse MI model (Figure 2) and H9c2 cells (Figure 3). UPLC-MS/MS profiling revealed 122 chemical compounds (Figure 4) from BYDE. Network pharmacology analysis predicted that BYDE could target 37 MI-related proteins in the treatment of MI. Further, pathway enrichment analysis suggested that BYDE might exhibit anti-inflammatory and cardioprotective activities through inhibiting the TNF-α/NF-κB pathway (Figure 5). Subsequently, the present study proposed and validated the four-compound mixture AGILe (i.e., adenosine, ginsenoside Rh2, isoliquiritigenin, and licochalcone A) for targeting the TNF-α/NF-κB pathway and protecting cardiomyocytes against OGD challenge (Figure 7).

TNF-α is one of the most potent pro-inflammatory cytokines, rapidly upregulated and increased the risk of recurrent coronary events in myocardial infarction (Ridker et al., 2000; Dorge et al., 2002; Gilles et al., 2003). The effects of TNF-α on the pathological remodeling and proinflammatory responses in heart failure are complex and highly receptor-specific (Ridker et al., 2000; Monden et al., 2007). Specifically, TNF-α receptor 1 (TNFR1) exacerbates the toxic effects, whereas TNFR2 mediates protective effects in various inflammatory diseases (Monden et al., 2007; Hamid et al., 2009). TNF-α is well-known to serve as a foundation cytokine and regulates the expression of other proinflammatory mediators including IL-1 and IL-6 *via* the NF-κB pathway (Aggarwal, 2003). On the other hand, NF-κB represents a family of inducible transcription factors that controls the initiation and progression of inflammation and provokes the infiltration and differentiation of immune cells (e.g., monocytes, macrophages, neutrophils) in infarct heart through inducing the expression of multiple target genes (Kis et al., 2003; Liu et al., 2017). Upon activation by various stimuli including oxidative stress, cytokines and infection, NF-κB not only regulates inflammation *via* promoting the production of inflammatory cytokines (e.g., IL1, IL6, IL8, TNF-α), chemokines (e.g., MCP-1, RANTES, CXCL1, CXCL10) and adhesion molecules (e.g., ICAM1, VCAM1, MMPs), but also affects the morphogenesis, apoptosis, proliferation and differentiation of the cells *via* altering the expression of various target genes (e.g., BCL2, BCL2L1, caspases, surviving, PAI2, cyclins) (Perkins, 2007; Madonna and De Caterina, 2012; Liu et al., 2017). NF-κB is activated through two major signaling pathways, the canonical or non-canonical pathways, to govern immune and inflammatory responses (Vallabhapurapu and Karin, 2009; Liu et al., 2017). The canonical NF-κB pathway mediates the intracellular signaling pathways of various cytokine receptors, pattern-recognition receptors, TNF receptor superfamily members (Liu et al., 2017). As for the canonical NF-κB activation, primarily, the inducible IκBα degradation allows rapid and transient nuclear translocation of canonical NF-κB members, predominantly the p50/RelA and p50/c-Rel dimers (Hayden and Ghosh, 2008; Liu et al., 2017).

In the present study, UPLC-MS/MS profiling identified 122 chemical compounds while network pharmacology analysis predicted 64 compounds for targeting 37 gene/protein targets. Pathway enrichment analysis identified 14 compounds for targeting 13 protein targets in relation with the TNF-α signaling pathway and 11 compounds for targeting 10 protein targets in relation with the NF-κB signaling pathway. Among these protein targets, five proteins (i.e., PTGS2, TNF, RELA, NFKB1, ICAM1) were commonly presented in both pathways. Among the compounds for targeting the TNF-α/NF-κB pathway, firstly, isoliquiritigenin was previously demonstrated for attenuating myocardial ischemia reperfusion injury and inhibiting the activity of TNF-α(Chi et al., 2017; Yushan et al., 2018; Yao et al., 2022). The present study found that isoliquiritigenin could target JUN, FOS, MMP9, and NFKB1 in the TNF-α pathway and CXCL12 and NFKB1 in the NF-κB pathway (Figure 5A). Secondly, licochalcone A not only directly inhibited the activation of the TNF-α/NF-κB pathway but also exhibited strong potential in the inhibition of angiotensin converting enzyme (ACE) in hypertension, heart failures and myocardial infarction (Funakoshi-Tago et al., 2009; Ke et al., 2017). The present study found that licochalcone A could target RELA and NFKB1 in the TNF-α pathway and CXCL12, RELA and NFKB1 in the NF-κB pathway (Figure 5A). Thirdly, several previous studies suggested adenosine as an adjunct therapy in ST elevation myocardial infarction patients (Kassimis et al., 2015; Aetesam-Ur-Rahman et al., 2021). Possibly, adenosine could induce inotropic effect on heart cultures and thereby protect cardiomyocytes against hypoxia (El-Ani et al., 2007). The present study found that adenosine mainly targeted PLCG1 in the NF-κB pathway (Figure 5A). Fourthly, ginsenoside Rh2 could inhibit NLRP3 inflammasome activation and alleviate hypoxia-induced myocardial injury possibly through the inhibition of NF-kB signaling (Lian et al., 2013; Yi et al., 2013; Qi et al., 2022). We could not exclude the importance of other chemical compounds in the cardioprotection and the regulation of the TNF-α/NF-κB pathway. Based on the contents and commercial availability of the chemical compounds and the relevance of the corresponding protein targets, four compounds (i.e., adenosine, ginsenoside Rh2, iso-liquiritigenin, licochalcone A) were selected for the preparation of the four-compound mixture AGILe. Our *in vitro* assays demonstrated that the mixture AGILe could effectively protect cardiomyocyte H9c2 cells against OGD challenge and decreased the levels of TNF-α, NF-κB and IkBa expression (Figure 7). It is noteworthy that BYDE is characterized by strong chemical diversity while different compounds in the formulation contribute to the bioactivity and toxicity. AGILe exhibited effects when four compounds were used the higher concentrations than that in BYDE. Nevertheless, these results suggested that better understanding on the composition and molecular targets of the traditional herbal medicines could facilitate the development of novel polychemical remedies against myocardial injury.

## Conclusion

The present study validated the cardioprotective effects of an in-house herbal medicine preparation BYDE in a mouse MI model, profiled 122 chemical compounds from BYDE by UPLC-MS/MS technology, predicted 37 proteins as the potential targets by network pharmacology analysis, and identified the preferable association with the TNF-α/NF-κB pathway. The deduced four-compound mixture AGILe was validated for the effects on the cell survival against OGD/R challenge and the activation of TNF-α/NF-κB pathways in H9c2 cells. The success of the present study not only proved the mixture AGILe as a new anti-MI therapy but also demonstrated a general approach for the development of novel polypharmacological formulations with defined chemical composition and specific mechanisms from complex herbal medicines.

## Data Availability

The original contributions presented in the study are included in the article/supplementary material, further inquiries can be directed to the corresponding author.
